# Hematuria: An uncommon presentation of Glanzmann's thrombasthenia—Lessons learnt

**DOI:** 10.4103/0970-1591.60456

**Published:** 2010

**Authors:** Sriram Krishnamoorthy, Santosh Kumar, Nitin Kekre

**Affiliations:** Department of Urology, Christian Medical College, Vellore, India

**Keywords:** Glanzmann's thrombasthenia, hematuria, platelet transfusions

## Abstract

A 55-year-old man with Glanzmann's thrombasthenia had recurrent episodes of gross painless hematuria for the past 30 years. His last episode of hematuria occurred a month ago, associated with pain in the right loin and was diagnosed to have a right mid-ureteric calculus. Under adequate platelet cover, he underwent right ureteroscopy. Postoperatively, he had persistent significant hematuria that did not improve despite repeated platelet transfusions. Factor VIIa was also transfused, without much benefit. A ureteroscopy was done, which identified bleeding from within the renal pelvis. CT angiogram confirmed the rupture of an artery supplying the interpole segment of the right kidney. Bleeding settled after angioembolization. Indiscriminate use of platelet transfusions would result in a state of platelet refractoriness. It is also important to suspect an iatrogenic cause for any complication that occurs after a surgical procedure, even if there could be an underlying medical etiology that can be attributed to the development of such complication.

## INTRODUCTION

Glanzmann's thrombasthenia (GT) is an autosomal recessive disorder. Although rare, it is a relatively more common platelet function defect, especially in communities where consanguinous marriages are more prevalent. The essential diagnostic features are a normal platelet count and morphology, a prolonged bleeding time and an absence of platelet aggregation.

Hematuria is an uncommon presentation of GT. Patients with bleeding disorders usually responds well to platelet transfusions. On the other hand, indiscriminate use of platelet rich concentrate might lead on to a state of refractoriness to further platelet therapy. This case report emphasizes that one should first suspect an iatrogenic reason for any complication that occurs after a surgical procedure, even if there could be an underlying medical etiology that can be attributed for the development of such a complication.

## CASE REPORT

A 55-year-old man, a known patient with Glanzmann's thrombasthenia (GT), was passing blood and blood clots in urine for the past 30 years. He had undergone a craniotomy and evacuation of a subdural hematoma in the past. He also gets recurrent episodes of epistaxis, which are managed conservatively.

**Figure 1:**
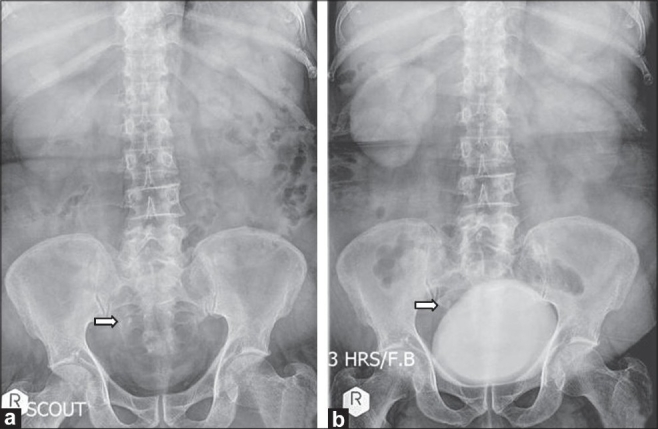
(a and b) Stone in the right mid ureter with a proximal hydroureteronephrosis

The last episode of hematuria occurred a month ago, and was associated with pain in the right loin. He was evaluated for the same and diagnosed to have a 1-cm right mid-ureteric calculus [Figure 1a and b]. Under adequate platelet cover, he underwent right ureteroscopy. Retrograde ureterogram revealed a 1 cm size stone at the mid-ureter, with a narrowing of the ureter of 6 Fr caliber. The stricture was dilated using a balloon dilator over a 0.035-inch polytetrafluoroethylene guidewire. The guidewire had accidentally perforated the interpole renal parenchyma, which was quickly recognized and withdrawn into the pelvicalyceal system. Ureteroscopy revealed an inflamed ureteral mucosa at the site of stone impaction. The ureter proximal to the stone was found to be grossly dilated with large amounts of altered blood and blood clots, thus obscuring vision. The stone was fragmented using Laser lithotripsy, leaving a double-J stent *in situ*.

Postoperatively, hematuria did not settle despite adequate platelet transfusions. Multiple blood transfusions were also given as the hemoglobin continued to decline. Factor VIIa was also transfused, with no benefit. As hematuria did not settle with conservative measures, a repeat ureteroscopy was done. There was a bloody efflux from the right ureteric orifice, with fresh bleeding from the pelvi-calyceal system.

**Figure 2:**
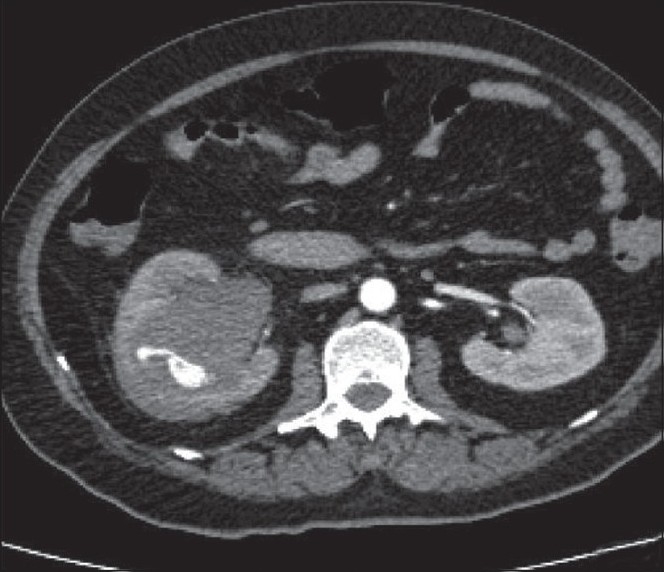
CT angiogram showing ruptured artery supplying the interpole

**Figure 3:**
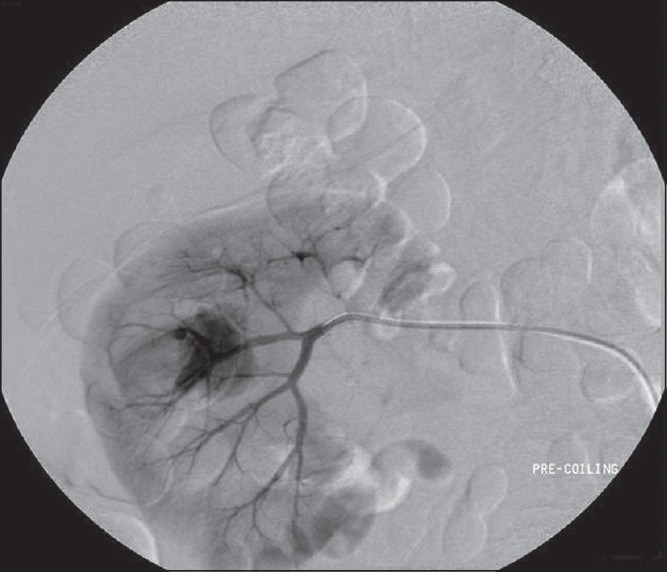
Renal angiogram confirming the ruptured artery

**Figure 4:**
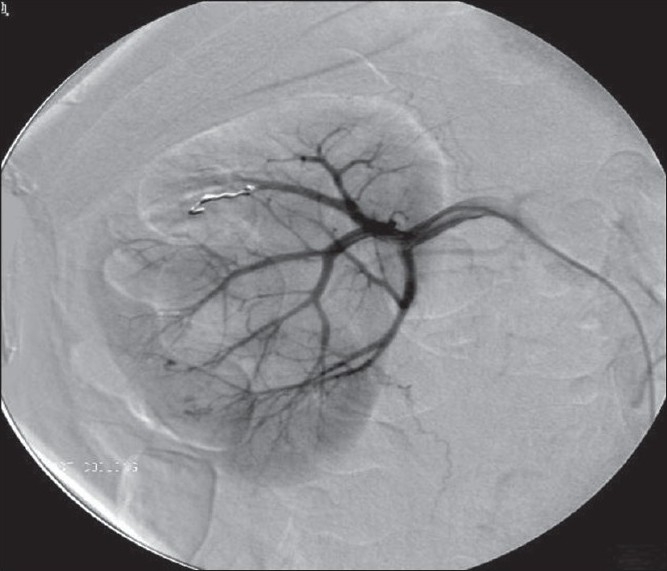
Postangioembolization renal angiogram

CT angiogram identified rupture of an artery supplying the interpole segment of the right kidney [Figure 2], which was further confirmed by a conventional angiogram [Figure 3]. Hematuria settled after angioembolization [Figure 4].

## DISCUSSION

Glanzmann's thrombasthenia (GT) is one of the rare autosomal recessive bleeding syndromes.[1] It affects the megakaryocytic lineage and is characterized by a lack of platelet aggregation, due to quantitative and/or qualitative abnormalities of ’αII3β integrin, which is a receptor that helps in aggregation of platelets at the site of any endothelial injury.[2] Glanzmann first described this disease in 1918 as “*hereditary hemorrhagic thrombasthenia*”.[3] It is a relatively more common platelet function defect in communities in which consanguineous marriages are more frequent.[4]

The clinical importance of this disease stems from the fact that GT is associated with varied clinical presentations. Some patients have only a minor bruising, while others have potentially fatal hemorrhages. The bleeding usually presents as purpura, epistaxis and menorrhagia or rarely as spontaneous hemothorax.[5] It can also present as gingival hemorrhage, as even a trivial trauma or pressure over the gums can lead to spontaneous bleeding.[6] The youngest ever-reported patient to have GT was diagnosed as early as sixth day of neonatal life.[7] Hematuria is a relatively uncommon manifestation, which usually settles with conservative measures. Only one case of GT has been reported in literature, requiring angioembolization to control hematuria.[8]

The severity of hematuria depends on the type of GT. Type I is characterized by an absence of GpIIb/IIIa complex, where there is an absence of platelet aggregation and clot retraction. In type II, there is a partial deficiency of GpIIb/IIIa complex, where aggregation is absent, but residual clot retraction occurs. In the third type (variant type), even though GpIIb/IIIa complex expression is >50% of the normal, since they are functionally impaired, these patients manifest with bleeding diathesis.[9]

It should be remembered that patients with inherited platelet disorders rarely need platelet transfusions. Even in patients with severe inherited platelet disorders such as GT, only sporadic bleeding occurs. They remain symptom-free for many years in between episodes. Our patient had his first episode of hematuria 30 years ago, but remained symptom-free for many years in between episodes. Patients with GT rarely need platelet transfusions. Most of the times, bleeding settles with conservative measures. In patients with massive bleeding, platelet-rich concentrates may be transfused. The likelihood of alloimmunization and subsequent refractoriness of platelets are the reasons why there should not be an indiscriminate use of platelet transfusions in GT. Recombinant factor VIIa has been shown to be very effective in such circumstances and also for prophylaxis in patients undergoing surgical procedures. Administration of factor VIIa to a patient with GT results in an enhancement of thrombin generation, which in turn increases the number of activated platelets deposited at the site of injury, thus increasing the available procoagulant surface.[10]

Our patient had an exacerbation of bleeding diathesis following ureteroscopic procedure, which was initially attributed to GT. He had received more than 30 units of platelet-rich concentrates and also factor VIIa. Because there was no improvement, he a relook ureteroscopy, which identified bleeding from the pelvi-calyceal system.

This report emphasizes that it is important for us to remember that one should suspect an iatrogenic reason for any complication that occurs after a surgical procedure, even if there could be an underlying medical etiology that can be attributed for the development of such a complication. Moreover, GT is an inherited disorder of platelets causing a defective platelet aggregation, leading to bleeding diathesis. Indiscriminate use of platelet-rich concentrate might lead to a state of refractoriness to further platelet therapy.
